# Recording of cardiovascular risk factors by general practitioners in patients with schizophrenia

**DOI:** 10.1186/s12991-020-00284-5

**Published:** 2020-05-19

**Authors:** Mª Carmen Castillejos, Carlos Martín-Pérez, Antonio García-Ruiz, Fermín Mayoral-Cleries, Berta Moreno-Küstner

**Affiliations:** 1 Department of Personality, Assessment and Psychological Treatment, Faculty of Psychology Andalusian Group of Psychosocial Research (GAP), University of Malaga, Campus Teatinos, 29071 Malaga, Spain; 2 North East Granada Sanitary District, Clinical Management Unit at Marquesado, Andalusian Health Service, Carretera los Pozos, 18518 Alquife, Granada Spain; 3 Department of Health Economics and the Rational Drug Use of Medicines. Faculty of Medicine, University of Malaga, Campus Teatinos, 29071 Malaga, Spain; 4 Clinical Management Unit of Mental Health of the Regional Hospital of Malaga. Andalusian Health Service, Biomedical Research Institute of Malaga (IBIMA), Plaza del Hospital, 29009 Malaga, Spain; 5 Department of Personality, Assessment and Psychological Treatment, Faculty of Psychology. Andalusian Group of Psychosocial Research (GAP). Biomedical Research Institute of Malaga (IBIMA), University of Malaga, Campus Teatinos, 29071 Malaga, Spain

**Keywords:** Cardiovascular disorders, Medical comorbidity, Mental health, Primary care, Severe mental illness

## Abstract

**Background:**

Patients with schizophrenia and related disorders (SRD) are more predisposed to having cardiovascular risk factors (CVRFs) compared to the general population due to a combination of lifestyle factors and exposure to antipsychotic medications. We aimed to analyse the documentation practices of CVRFs by general practitioners (GPs) and its associations with patient variables in a sample of persons with SRD.

**Methods:**

An observational, cross-sectional study was conducted in 13 primary care centres (PCCs) in Malaga (Spain). The population comprised all patients with SRD who were in contact with a GP residing in the study area. The number of CVRFs (type 2 diabetes mellitus, hypertension, hypercholesterolaemia, obesity and smoking) recorded by GPs were analysed by considering patients’ demographic and clinical variables and use of primary care services. We performed descriptive, bivariate and multivariate regression analyses.

**Results:**

A total of 494 patients were included; CVRFs were not recorded in 59.7% of the patients. One CVRF was recorded in 42.1% of patients and two or more CVRFs were recorded in 16.1% of patients. Older age, living in an urban area and a higher number of visits to the GP were associated with a higher number of CVRFs recorded.

**Conclusion:**

The main finding in this study is that both patients’ demographic variables as well as use of primary care services were found to be related to the documentation of CVRFs in patients with SRD by GPs.

## Background

It has long been accepted that patients with severe mental illness (SMI) have higher rates of mortality and morbidity from physical health problems compared to the general population [1–3], due to the high level of CVRFs they present [4]. A meta-analysis performed by Osborn et al. [5] revealed that these patients presented a pooled risk ratio of 1.70 (95% confidence interval [CI] 1.21–2.37) for diabetes and 1.11 (95% CI 0.91–1.35) for hypertension. In a further study carried out in London [5], patients with schizophrenia exhibited greater risk of developing CVRF, such as diabetes, hypertension, hyperlipidaemia, smoking and obesity. These results are congruent with many other studies [6–11].

Nevertheless, little attention continues to be paid to the physical problems of patients with SMI, especially considering that these individuals are at risk of underdiagnosis and under-treatment [12]. Smith et al. [3] reported that most people with schizophrenia had at least one chronic physical comorbidity and one-third had two or more. However, cardiovascular disorder comorbidities among these patients were under-recorded relative to individuals without schizophrenia. Woodhead et al. [13] found similar results, notwithstanding the fact that the number of consultations with primary care was high. Additionally, patients with SMI receive fewer prescriptions for the treatment of common CVRFs [14]. This phenomenon could be because most health professionals consider the care of people with SMI too specialised for primary care [15].

Patients with SRD are more predisposed to having CVRFs compared to the general population due to a combination of lifestyle factors and exposure to antipsychotic medications. We hypothesise that there may be a relationship between the number of CVRFs recorded in the clinical records of patients with SRD by GPs and patients’ demographic and clinical variables and use of primary care services variables. We aimed to examine the documentation practices of CVRFs (type 2 diabetes mellitus, hypertension, hypercholesterolaemia, obesity and smoking) by GPs in patients with SRD and their association with patients’ demographic and clinical variables and use of primary care services.

## Methods

An observational, cross-sectional study was conducted.

### Setting of the study

This study was implemented in the area of the Clinical Management Unit of Mental Health (CMU-MH) of the Regional Hospital of Malaga, whose population coverage comprises 315,159 inhabitants. This area includes two community mental health units (CMHU: Centre and North), which contain 13 primary care centres (PCCs). The study information was collected from 1 January, 2008 to 1 July, 2011.

### Patient and public involvement

The Malaga Schizophrenia Case Registry (RESMA) is a case registry of patients with SRD who attend the CMU-MH of the Regional Hospital of Malaga to improve the care of patients with SMI [16].

The inclusion criteria were patients: (a) aged 15 and older; (b) with a clinical diagnosis of SRD according to the International Classification of Diseases (ICD-10); and (c) in contact with a PCC in the coverage area of the CMU-MH of the Regional Hospital of Malaga. The exclusion criteria were patients: a) treated outside the study area; b) who died during the follow-up; and c) not having a computerised medical record at the reference PCC [17].

The eligible population comprised all patients included in the RESMA (*N* = 1663) [18], from which we selected via simple random sampling a representative sample of all PCCs. A total of 528 patients were selected, but 34 (6%) did not have a digitalised medical record in primary care, and so were not included in the study. Ultimately, a total of 494 patients were included.

### Study variables

Our dependent variable was the number of CVRFs (type 2 diabetes mellitus, hypertension, hypercholesterolaemia, obesity and smoking) recorded by the SRD patients’ GPs. The independent variables were: (1) demographic factors: gender (male or female); age (15–44, 45–64 or > 65 years); marital status (single, married/civil partnership/cohabiting or separated/divorced/widowed); educational level (no formal education and/or illiterate, primary school, secondary school or higher education [bachelor’s degree or higher]); living arrangements (alone, living with parents/other relatives or friends, own family, sheltered accommodation or homeless); employment status (employed, unemployed, student, carer or househusband/housewife, receiving welfare benefits or temporary work disability); type of living area (urban or rural); living within a socioeconomically deprived area (no or yes); (2) clinical factors: ICD-10 clinical diagnosis (F20 schizophrenia, F22 persistent delusional disorders, F23 acute and transient psychotic disorders, F25 schizoaffective disorders, F21 schizotypal disorder, F24 induced delusional disorder, F28 other non-organic psychotic disorders or F29 unspecified non-organic psychosis); Global Level of Severity (GLS; level I: low severity, level II: medium severity, level III: high severity); and (3) use of primary care services: number of visits to GP; number of visits to nurse; total number of visits to PCC (including GPs and nurses).

The GLS is an index assigned to patients with SRD by psychiatrists, who usually treat patients according to symptom severity, disease evolution, social adjustment, disability level and treatment adherence. This index is used by psychiatrists in the clinical setting of public mental health services in order to include patients within the category of “Severe Mental Illness” and thus access additional resources [19].

### Data sources

Data on patients’ number of CVRFs and visits to their GP, nurse and PCC were obtained from digitised primary care records (DIRAYA programme). Patients’ demographic and clinical information were collected from the RESMA [16, 18].

### Data analysis

The categorical variables used in the descriptive analysis were reported using frequency distribution and percentages. Descriptive statistics (mean, standard deviation, median and quantiles) were calculated for the continuous variables.

We used bivariate analysis to examine the relationships between the dependent variable (CVRFs) and the independent variables. To study the association between the dependent variable and the categorical explanatory variables with two categories, a non-parametric test (Wilcoxon) was used. In the case of a categorical explanatory variable that was continuous or had more than two categories, we used simple linear regression.

Finally, a linear regression was adjusted. In the first step, an initial model was adjusted with all the explanatory variables. In the second step, the variables that did not reach statistical significance were removed from the model; this did not imply substantial changes in the coefficients of the remainder of the variables. Differences were considered significant at *p* < 0.05.

### Results

The database comprised 494 patient records, of which 70% were men. The mean age at the beginning of the study was 43.8 years (SD 11.64). The majority of patients were single (70.6%). Most of the patients had not completed secondary school (64.4%). The most common living arrangements comprised living with parents, other relatives or friends (56.9%). As regards patients’ work situations, 42.9% were unable to work. Patients predominantly lived in an urban environment (87.1%). Only 11.5% of the sample resided in socioeconomically deprived areas (Table 1).

**Table 1:** Sociodemographic and clinical characteristics of patients with schizophrenia and related disorders (*N* = 494)

Sociodemographic characteristics	Patients *N* (%)
Gender	
Male	327 (66.3%)
Female	167 (33.8%)
Age	
15–44	265 (53.6%)
45–64	205 (41.5%)
> 65	24 (4.9%)
Marital status	
Single	349 (70.6%)
Married/civil partnership/cohabiting	90 (18.2%)
Separated/divorced/widowed	55 (11.1%)
Educational level	
No formal education and/or illiterate	81 (16.4%)
Primary school	237 (48%)
Secondary school	126 (25.5%)
Higher education (bachelor’s degree)	50 (10.1%)
Living arrangements	
Alone	54 (10.9%)
With parents/other relatives or friends	281 (56.9%)
Own family	102 (20.6%)
Sheltered accommodation	52 (10.5%)
Homeless	5 (1.0%)
Employment status	
Employed	76 (15.4%)
Unemployed	85 (17.2%)
Student	29 (5.9%)
Carer or househusband/housewife	30 (6.1%)
Receiving welfare benefits	212 (42.9%)
Temporary work disability	62 (12.6%)
Type of living area	
Urban	430 (87.1%)
Rural	64 (12.9%)
Living within a socioeconomically deprived area	
No	437 (88.5%)
Yes	57 (11.5%)
Clinical characteristics	
ICD-10 clinical diagnosis	
F20 Schizophrenia	346 (70%)
F22 Persistent delusional disorders	53 (10.7%)
F23 Acute and transient psychotic disorders	46 (7.3%)
F25 Schizoaffective disorders	36 (9.3%)
F21, F24, F28, F29 Schizotypal disorder, Induced delusional disorder, other non-organic psychotic disorders and unspecified non-organic psychosis	13 (2.6%)
Global level of severity	
Level I (low severity)	157 (31.8%)
Level II	189 (38.2%)
Level III (high severity) (Missing data:63)	85 (17.2%)
Total	494 (100%)

The most frequent diagnosis was schizophrenia (70.04%), followed by persistent delusional disorders (10.7%), schizoaffective disorder (9.3%), acute and transient psychotic disorders (7.3%) and, finally, 2.6% of patients presented other diagnoses. More than a third of patients presented level II severity (38.2%; Table 1).

During the study period, the mean number of patients’ visits to their PCC was 22.25 (SD 21.32; 0–100), mean number of visits to their GP was 14.35 (SD 12.45; 5–75) and mean number of visits to their nurse was 7.75 (SD 14.89; 2–93; Table 2).

**Table 2:** Bivariate analysis of the number of cardiovascular risk factors recorded in patients with schizophrenia and related disorders (*N* = 494)

	W Wilconxon	Estimate	Standar error	*t* value	*p* value
Gender	30202.5				0.028
Male					
Female					
Age		0.024	0.004	6.757	*p* < 0.001
Marital status					
Single		Reference			
Married/Civil partnership/Cohabiting		0.339	0.111	3.055	0.002
Separated/Divorced/Widowed		0.250	0.136	1.836	0.067
Educational level					
No formal education and/or illiterate		Reference			
Primary school		− 0.114	0.121	− 0.945	0.345
Secondary school		− 0.307	0.134	− 2.291	0.022
Higher education (Bachelor’s degree)		− 0.415	0.169	− 2.452	0.015
Living arrangements					
Homeless		Reference			
Alone		0.522	0.439	1.189	0.235
With parents / other relatives or friends		0.330	0.424	0.779	0.436
Own family		0.643	0.430	1.494	0.136
Sheltered accommodation		0.588	0.440	1.337	0.182
Employment status					
Receiving welfare benefits		Reference			
Employed		− 0.358	0.124	− 2.889	0.004
Unemployed		− 0.421	0.124	− 3.541	*p *< 0.001
Student		− 0.465	0.183	− 2.537	0.012
Carer or househusband/housewife		0.122	0.181	0.678	0.498
Temporary work disability		− 0.457	0.134	− 3.421	*p *< 0.001
Type of area	16388.5				0.005
Urban					
Rural					
Living within a socioeconomically deprived area	10690				0.048
Yes					
No					
ICD-10 clinical diagnosis					
F20 schizophrenia		Reference			
F21, F24, F28, F29 schizotypal disorder, induced delusional disorder, other non-organic psychotic disorders and unspecified non-organic psychosis		− 0.274	0.268	− 1.024	0.306
F22 persistent delusional disorders		− 0.036	0.140	− 0.260	0.795
F23 acute and transient psychotic disorders		− 0.159	0.149	− 1.068	0.286
F25 schizoaffective disorders		0.091	0.166	0.548	0.584
Global level of severity					
Level III (high severity)		Reference			
Level II		0.183	0.126	1.455	0.147
Level I (low severity)		0.283	0.130	2.180	0.030
Number of visits to primary care centre		0.015	0.002	8.129	*p *< 0.001
Number of visits to general practitioner		0.026	0.003	8.140	*p *< 0.001
Number of visits to nurse		0.013	0.003	4.660	*p *< 0.001

Regarding patients’ number of recorded CVRFs, 59.7% did not present any recorded CVRFs, 42.1% presented one recorded CVRF and 16.1% presented two or more. Type 2 diabetes mellitus was recorded in 10.5% of patients, hypertension in 13.2%, dyslipidaemia in 15.8% and obesity in 10.3%; 14.2% were smokers.

### Factors associated with the number of documented CVRFs

The bivariate analysis indicated that the factors associated with a lower number of recorded CVRFs were: being male (*p* = 0.028), residing in rural areas (*p* = 0.005), having secondary (*p* = 0.022) or higher (*p* = 0.015) levels of education and being employed (*p* = 0.004), unemployed (*p* < 0.001), students (*p* = 0.012) or on temporary work disability (*p* < 0.001) compared with receiving welfare benefits. On the other hand, the factors that were associated with a greater number of recorded CVRFs were: having a partner (*p* = 0.002), being older (*p* < 0.001), residing in socioeconomically deprived areas (*p* = 0.048), presenting a level I illness severity (*p* = 0.030) and increased number of visits to the GP, nurse and PCC (*p* < 0.001). There were no significant differences in the type of living arrangement and diagnosis categories (Table 2).

The multivariate linear model revealed that there were five variables that presented a clear association with the number of CVRFs recorded by GPs: age, living arrangements, type of living area, employment status and number of visits to the PCC. Older age was associated with a greater number of recorded CVRFs (*p* < 0.001). Patients who had their own family (*p* = 0.049) and those who lived in sheltered accommodation (*p* = 0.048) presented more recorded CVRFs compared with homeless patients. The higher the number of patient’s visits to the PCC, the greater the number of CVRFs recorded (*p* < 0.001). On the other hand, fewer CVRFs were recorded in patients living in rural areas (*p* = 0.028). For the employment category, only the group classified as temporary work disability (*p* = 0.022) had fewer CVRFs recorded compared to those who were receiving welfare benefits (Table 3).

**Table 3:** Multilevel linear regression of the number of cardiovascular risk factors recorded in patients with schizophrenia and related disorders (= 494)

	Estimate	Standar Error	*t* value	*p* value
Age	0.017	0.004	4.474	*p* < 0.001
Living arrangements				
Homeless	Reference			
Alone	0.720	0.402	1.791	0.074
With parents/other relatives or friends	0.706	0.390	1.812	0.071
Own family	0.781	0.395	1.975	0.049
Sheltered accommodation	0.798	0.403	1.983	0.048
Employment status				
Receiving welfare benefits	Reference			
Employed	− 0.167	0.121	− 0.606	0.168
Unemployed	− 0.172	0.117	− 1.476	0.141
Student	− 0.108	0.178	− 0.606	0.545
Carer or househusband/housewife	0.029	0.172	0.165	0.869
Temporary work disability	− 0.290	0.126	− 2.302	0.022
Type of living area				
Urban	Reference			
Rural	− 0.255	0.116	− 2.200	0.028
Number of visits to primary care centre	0.013	0.002	7.204	*p* < 0.001

## Discussion

This study examined the recording practices by GPs of CVRFs of patients with SRD. The novelty of this study is that we were able to analyse a large number of patients with SRD in a primary care setting.

The percentages of CVRFs recorded in our sample were lower than those obtained for the general Andalusian population [20]. This result is unexpected given that the scientific literature describes SRD patients as being more predisposed to having multiple CVRFs [4]. Interestingly, similar results to the current study were reported in a Spanish study by Viñas et al. [21]. In Spain, all patients are assigned a GP, who represents the gateway to the Spanish health system and can refer patients to specialists. Thus, all patients are first treated by their GP, who refers them to a psychiatrist or other specialist if and when necessary. Viñas et al. explained their results as owing to these patients having limited access to the health system, hence they could not benefit from the preventative initiatives offered to the general population. Additionally, there was a lack of coordination between GPs and psychiatrists in patient care [21]. Bernardo et al. [22] found that factors such as smoking, alcoholism and excess weight were easily recognisable by doctors. However, factors such as dyslipidaemia were underdiagnosed, and a sedentary lifestyle was not recognised as a risk factor. Further studies may be necessary in this area for a reliable comparison of the documentation of CVRFs in patients with SRD by GPs with the same documentation in control patients. Moreover, it would also be interesting to compare the documentation of CVRFs in patients with SRD by GPs with the same data recorded by physicians other than GPs.

Regarding gender, bivariate analysis found that a greater number of CVRFs were recorded in women, coinciding with the results found by Baldiseroto et al. in the general population of Brazil [23]. However, Rahman et al. found no gender differences in a study conducted in the general population of Bangladesh [24]. Both bivariate analysis and the multivariate linear model found that the older the patient, the greater the number of registered CVRFs. This finding is consistent with the majority of studies performed in the general population [23–29]. In the bivariate analysis, it was shown that a greater number of CVRFs were recorded in patients who had a partner; however, these differences did not appear in the multivariate linear model. Baldiseroto et al. [23] found a higher prevalence of CVRFs in people with a partner; however, Rahman et al. [24] found that these people had the least prevalence. In two more studies that analysed this variable, no differences were found in the prevalence of CVRFs in the general population [28, 29]. Regarding educational level, only bivariate analysis showed significant differences, finding that patients with the highest educational level presented the least number of CVRFs. Comparing our results with other studies, Rahman et al. [24] found no significant differences in the prevalence of CVRFs between different educational levels, however, the study carried out in Brazil by Baldiseroto et al. [23] found similar results to ours. Interestingly, there were several studies that analysed this variable by gender, finding a lower prevalence of CVRFs at higher educational levels, but only in women [26, 28–31]. The multivariate linear model showed that homeless patients had fewer CVRFs recorded than other patients. We did not find any study that compared this variable with the number of CVRFs. Bivariate analysis showed that, with respect to patients receiving welfare benefits, patients who were in other work situations (except housewives) had less CVRFs recorded. This result is similar to that found in a study carried out by Gazhali et al. in Malaysia, in which a greater number of CVRFs were identified among housewives [29]. This was due to the high prevalence of obesity, low fruit and vegetable consumption and physical inactivity [29]. Another study carried out in the Philippines found that the prevalence of CVRFs was highest among the self-employed and lowest among individuals with irregular employment [27]. This could be related to the fact that in the same study it was found that individuals with the highest level of education had the highest prevalence of CVRFs, and perhaps these individuals did not carry out irregular work. However, the multivariate linear model showed significant differences only in patients with temporary work disability compared to those who received welfare benefits or pensions. Contrary to our results, a study of the general population of Catalonia (Spain) identified no differences in the record of CVRFs in terms of patients’ employment status [25]. In addition, more CVRFs were recorded in urban areas, similar to findings attained in the general Polish population [30] and in developing countries [27, 29]. By contrast, a study conducted in the USA by Trivedi et al. found a higher prevalence of metabolic syndrome in rural compared to urban settings [31]. Bivariate analysis showed a higher number of CVRFs in patients living within a socioeconomically deprived area and, regarding the global level of severity, the most severely affected patients had less CVRFs than the less severely affected. However, we did not find any study that compares these variables with the prevalence of CVRFs. There was no significant difference between ICD-10 codes for the number of recorded CVRFs. Finally, this model showed that more visits to primary care services were associated with an increased number of recorded CVRFs. This relationship seems logical because the more contact a patient has with health services, the greater the potential for their physical comorbidities to be recorded.

### Strengths and limitations of this study

#### Limitations

- This report was a cross-sectional study, from which we could only infer association and not causality.
- The study lacked a control group of patients selected from the same GP practices who were not diagnosed with significant mental illness.
- We were unable to obtain data on the prevalence of CVRFs in the general population. However, we obtained data on the prevalence of CVRFs in the general population of the same area in previous studies.
- Clinical diagnoses were not made through clinical interviews, but by the patients’ long-term psychiatrists and updated in the RESMA database.
- We selected patients diagnosed with SRD and we have not taken into account the evolution and the course of the disorder. However, most of the patients who had CVRFs recorded had a stable course of the illness.
- Although almost all patients were being treated with antipsychotic medications, we did not take into account the dose and the type of drugs in the clinical variables analysed.
- Information about patients was collected from medical records instead of having individual patients undergo specific health screening tests.
- The study excluded patients who died during the follow-up. This may have led to an underestimation of the cardiovascular events in the study sample.

#### Strengths

- We analysed a large number of patients with SRD in a primary care setting.
- The study was performed in a wide catchment area, including 13 PCCs.
- This study represents information from real clinical practice.

### Conclusions

This study demonstrated that some patients’ demographic variables as well as use of primary care services were associated with the CVRF records compiled by patients’ GPs. Given that patients with SMI have higher rates of mortality and morbidity from physical health problems compared to the general population, greater effort to document these risk factors in patients with SRD should be made by GPs.

## Data Availability

The data that support the findings of this study are available from Berta Moreno Küstner, but restrictions apply to the availability of these data, which were used under license for the current study, and so are not publicly available. Data are, however, available from the authors upon reasonable request and with permission of Berta Moreno Küstner.

## Abbreviations

CI: Confidence interval
CMU-MH: Clinical management unit of mental health
CVRFs: Cardiovascular risk factors
GLS: Global level of severity
GP: General practitioner
ICD: International classification of diseases
PCC: Primary care centre
RESMA: Malaga schizophrenia case registry
SMI: Severe mental illness
SRD: Schizophrenia and related disorders
